# Study on the Electrochemical Removal Mechanism of Oxytetracycline by a Ti/IrO_2_-Ta_2_O_5_ Plate

**DOI:** 10.3390/ijerph18041708

**Published:** 2021-02-10

**Authors:** Yinghao Zhang, Wenqiang Jiang, Hao Dong, Xuyang Hu, Baihui Fang, Guangfei Gao, Rui Zhao

**Affiliations:** College of Environmental Science and Engineering, Qilu University of Technology (Shandong Academy of Sciences), Jinan 250353, China; 17862975977@163.com (Y.Z.); 17854118848@163.com (H.D.); huxuyang1124@163.com (X.H.); fbh2145@126.com (B.F.); qluggf2020@163.com (G.G.); m13678823371@163.com (R.Z.)

**Keywords:** Ti/IrO_2_-Ta_2_O_5_ anode, oxytetracycline, electrochemical, mechanism

## Abstract

In this study, a Ti/IrO_2_-Ta_2_O_5_ anode was prepared by a hydrothermal method, and the prepared electrode was characterized by techniques such as scanning electron microscopy, X-ray diffraction, and electron dispersive spectroscopy. At the same time, the anode characteristics before and after electrochemical experiments were analyzed. The electrode gradation mechanism of oxytetracycline is discussed. In the whole experimental process, the range of electrolysis conditions was determined by single factor experiment, and then the optimal removal condition of oxytetracycline was determined by orthogonal experiments. The removal rate of oxytetracycline reached 99.02% after 20 min of electrolysis under the following optimal conditions: a current of 0.500 A, plate spacing of 2 cm, Na_2_SO_4_ electrolyte concentration of 4 g/L, and solution pH of 3. Additionally, the mechanism of oxytetracycline removal was explored, free radical scavenging experiments were performed, and the degradation mechanism was inferred based on the changes in the ultraviolet absorption of the oxytetracycline solution before and after electrolysis. Then, based on the liquid chromatography–mass spectrometry data, seven possible compounds and five possible removal pathways were proposed.

## 1. Introduction

In recent years, reports of the detection of antibiotics in various river basins have exposed the environmental problems arising from the use of antibiotics; this has motivated an increasing research effort focusing on the treatment of antibiotics [1,2]. Tetracycline antibiotics are currently some of the most widely and commonly used antibiotics. These antibiotics are widely used in the prevention and control of human diseases, agricultural production, and livestock and poultry breeding [3,4]. Oxytetracycline is the most representative tetracycline antibiotic that inhibits bacterial growth by inhibiting protein synthesis, and therefore has a good anti-infection effect, eliminating inflammation and promoting the growth of livestock [5]. Due to their stable chemical properties, antibiotics cannot be decomposed and absorbed by the liver and kidney after entering the organism and are mostly excreted by the kidney in the form of prototype drugs [6]. As a feed additive, they are directly added to aquaculture water. Microorganisms in the water can become resistant to the added antibiotics after long-term exposure to them in the environment, affecting the microbial community and aquatic organisms and destroying the aquatic ecosystem [7,8]. Additionally, antibiotics from various sources are eventually transported to water bodies, seep into groundwater, and contaminate drinking and tap water [9]. In addition, antibiotics remaining in water bodies and deposited in sediment re-enter the soil through irrigation and sludge fertilization and are absorbed into the food chain by microorganisms, algae, and crops. Through the food chain, antibiotics can accumulate in the human body over time and cause health problems [10,11].

There are no clear standards for the treatment of antibiotic wastewater in sewage treatment plants, and it is urgent to establish environmental standards that meet health requirements [12,13]. Conventional water treatment techniques, such as biological, physical, and chemical methods, have poor efficiency for the removal of antibiotics [14]. The long-term accumulation of antibiotics can easily cause serious environmental problems. Thus, advanced sewage treatment technologies should be introduced to address the challenges posed by industries showing high antibiotic pollution levels, such as aquaculture [15]. Zhao et al. studied the degradation mechanism of oxytetracycline under visible light and sunlight conditions, and found that the degradation rate of oxytetracycline reached 72% when titanium dioxide doped with nitrogen and fluorine was used as the catalyst [16]. Arikan et al. studied the degradation efficiency of oxytetracycline under different manure composting methods, and achieved a removal rate of 75% [17]. Li et al. studied a new material, ZIF-8, for the adsorption of oxytetracycline in water, achieving a removal rate of 82.5% [18]. Uslu and Balcioglu treated oxytetracycline wastewater with an electrochemical combination of Fenton and ozone oxidation, and obtained a removal rate of 93% [19]. As an emerging water treatment technology, electrochemical advanced oxidation technology has been increasingly studied due to its wide applicability and high efficiency [20]. Fernandes et al. used a boron-doped diamond electrode as an anode and successfully performed anodization of oxytetracycline [21]. The oxidation–reduction reactions that occur during the electrochemical degradation of organic matter are divided into direct reactions and indirect reactions [22]. Under the action of an electric field, large molecules and long chains are transformed into more easily biodegradable molecules that can be removed from water by aquatic microorganisms [23].

The electrochemical degradation reactions of antibiotics are divided into direct and indirect oxidation reactions. Direct oxidation reactions occur on the electrode surface through the electron transfer between the organic matter and the anode, while the indirect oxidation reaction is the oxidative degradation of organic matter promoted by the active groups (superoxide radicals, hydrogen peroxide, hydroxyl radicals, etc.) generated due to the electrode potential [24]. These groups have strong oxidizing ability, so that refractory organic pollutants that are difficult to treat by other methods can also be oxidized or even degraded to inorganic substances, such as water and carbon dioxide [25].

A coating material influences the life of a plate and the degree of mineralization and rate of degradation of the target pollutants. Currently, titanium plate substrates coated with inert metal plates are the most widely used plates. Due to the high oxygen absorption potential and resistance to acidic corrosion as an inert substance with strong stability, Ir-Ta oxide provides good protection for the coated electrode. Therefore, a Ti substrate coated with an Ir-Ta metal oxide coating was selected as the experimental electrode to study the degradation process of oxytetracycline in water.

## 2. Materials and Methods

### 2.1. Experimental Equipment and Materials

**Figure 1:**
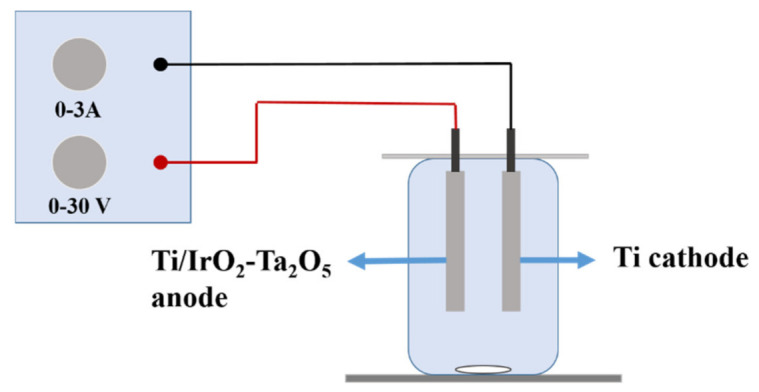
Schematic diagram of the experimental electrolytic apparatus.

The experimental equipment consisted of a DC power supply (KPS-3005DU, Shenzhen Zhaoxin Electronic Instrument Equipment Co., Ltd., China), electrolytic cell, electromagnetic stirrer (78-1, Jintan Medical Instrument Factory, China), Ti/IrO_2_-Ta_2_O_5_ anode, and Ti cathode. The electrolytic cell was a glass beaker with a capacity of 500 mL. The dimensions of the Ti/IrO_2_-Ta_2_O_5_ anode were 50 mm × 100 mm × 1 mm, and the dimensions of the Ti cathode were 50 mm × 100 mm × 1 mm. To achieve different current densities in this experiment, stable current output of the DC power supply was provided. The electromagnetic agitator accelerated the movement of simulated wastewater and the efficiency of electrolytic oxidation, and its rotational speed did not change as a quantitative factor. The experimental setup is shown in Figure 1.

### 2.2. Electrolytic Experiment Scheme

In the experimental simulation of the degradation, an electrolytic aqueous solution was prepared using oxytetracycline (20 mg) dissolved in deionized water (1 L). Oxytetracycline aqueous solution (400 mL) was added into a beaker with a capacity of 500 mL. Then, Na_2_SO_4_ was added to the electrolytic aqueous solution as the electrolyte. Experimental oxytetracycline (C_22_H_24_N_2_O_9_, USP Grade) was purchased from Shanghai Yuanye Biological Co., Ltd. (Shanghai, China). Single-factor electrolysis experiments for oxytetracycline wastewater were carried out first. The concentration of oxytetracycline was determined by HPLC at 5, 10, 15, and 20 min using 0.45 µm microporous membrane filtration sampling. The removal effect of oxytetracycline was analyzed for the plate spacings of 1, 2, 3, and 4 cm, current intensities of 0.1, 0.25, 0.4, and 0.55 A, electrolyte concentrations of 2, 3, 4, and 5 g/L, and pH values of the solutions of 3, 5, 7, and 9. After the data were analyzed, orthogonal experiments were carried out to determine the best experimental conditions, and the degradation process was analyzed on this basis. The DC power supply provided different current densities in the experiment. To meet the condition of setting different plate spacings in the experiment, insulating rulers with different spacings were designed to fix the cathode and anode plates. In addition, the pH of the electrolytic wastewater was regulated using NaOH and H_2_SO_4_.

### 2.3. Preparation of Electrodes

The Ti/IrO_2_-Ta_2_O_5_ anode was prepared by spraying an Ir-Ta oxide solution on the surface of a titanium plate. The process for the preparation of the Ti/IrO_2_-Ta_2_O_5_ anode was introduced and described in detail by Xu et al. [26]. The microstructure of the Ti/IrO_2_-Ta_2_O_5_ anode surface was observed by scanning electron microscopy (SEM) (JSM7800F). The microstructure of the Ti/IrO_2_-Ta_2_O_5_ anode surface was observed by SEM, and the composition of the electrode coating was analyzed by X-ray energy-dispersive spectroscopy (EDS). X-ray diffraction (XRD) was conducted with the material, and the diffraction pattern was analyzed to further determine the composition of the plate material along with the internal atomic or molecular structure.

### 2.4. Analysis Method

The concentration of oxytetracycline was determined by high-performance liquid chromatography (HPLC, LC-10Atvp, Shimadzu, Japan) with an InertSustain C18 (4.6 mm × 150 mm, 5 μm, filled with octadecylsilane bonded silica gel) column. Chromatographic conditions were as follows: the mobile phase was methanol–purified water−2% triethylamine (V/V/V = 100:100:1), using 10% glacial acetic acid to adjust the pH to 5–6; flow rate: 1.0 mL; column temperature: 25 °C; sample size: 20 μL; wavelength: 280 nm. The intermediate was determined by liquid chromatography using a mass spectrometer (TU-1900, Beijing General instrument Co., Ltd., Beijing, China) with an InertSustain C18 column, and the chromatographic detection conditions were as follows: mobile phase was methanol–deionized water (V/V = 500:500), pH was 5–6 adjusted with formic acid, flow rate was 0.5 mL/min, detection wavelength was set at 280 nm and 355 nm, sample size was 10 μL. The mass spectrometric conditions were as follows: the ion source was an electrospray ionization source (ESI), positive ion detection mode scanning, ion source temperature was 300 °C, mass charge ratio scanning range was 200~2800. In other experiments, the degradation process of oxytetracycline was determined by ultraviolet spectrophotometry (TU-1900, Beijing General Analysis Instrument Co., Ltd., Beijing, China).

### 2.5. Electrolytic Data Analysis

The kinetic equation of the electrochemical oxidation of oxytetracycline was expressed according to the pseudo-first-order kinetic model, as shown in Equation (1):

(1) $$-\frac{d_{c}}{d_{t}}=k_{a}c_{0}$$

where *c* (mg/L) is the concentration of oxytetracycline at time *t* (min), *c*_0_ (mg/L) is the initial concentration of oxytetracycline, and *k_a_* (min^−1^) corresponds to the first-order rate constant.

The concentration of oxytetracycline can be calculated according to the standard curve of oxytetracycline, and the degradation rate of oxytetracycline is formulated as shown in Equation (2):

(2) $$\eta =\left(c_{0}-c_{t}\right)\times 100\%$$

where η is the degradation rate (%) of oxytetracycline hydrochloride, *c*_0_ is the initial concentration (mg/L) of oxytetracycline hydrochloride, and *c_t_* is the concentration (mg/L) of oxytetracycline hydrochloride after *t* minutes of reaction time.

## 3. Results and Discussion

### 3.1. Factors Influencing the Degradation Effect of Oxytetracycline

**Figure 2:**
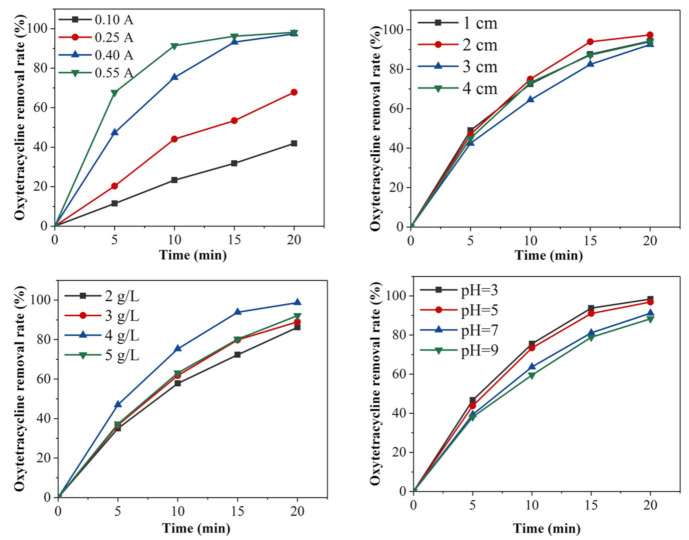
Degradation efficiency of oxytetracycline at different current densities/electrode spacings/electrolyte concentrations/solution pH values.

To explore the key factors affecting the electrochemical oxidation performance of oxytetracycline, the four key factors (current density, plate spacing, electrolyte concentration, and solution pH) were changed, and the control variable method was adopted for the experiment. Under experimental conditions, the removal of oxytetracycline followed quasi-first-order kinetics, but the observed removal efficiency varied, as shown in Figure 2. The experimental data were obtained by taking the average of three experiments, and the accuracy of the data was within the allowable range of error.

The current intensity affects the transfer rate of the electrons between the electrode and the solution, but a larger current incurs greater energy consumption, so it is important to choose a current that achieves good removal efficiency together with low energy consumption. To explore the effect of the current intensity on the removal of oxytetracycline, experiments were carried out by changing the current intensity (0.100, 0.250, 0.400, 0.550 A), while other conditions (plate spacing: 2 cm, Na_2_SO_4_ electrolyte concentration: 4 g/L, solution pH: 3) remained unchanged. As shown in Figure 2, for the current values of 0.1 and 0.25 A, the removal rate of oxytetracycline was 41.9 and 67.8% at 20 min, respectively, and for a current of 0.55 A, the removal rate of oxytetracycline reached 97.39% at 20 min. Thus, the removal rate of oxytetracycline in solution increased with increasing electric current. When the current increased, the electron transfer rate between the electrode and solution increased, and with an increased electron transfer, a large number of strongly oxidizing hydroxyl radicals were produced. Hydroxyl radicals with strong oxidizing ability react with oxytetracycline, and the removal rate of oxytetracycline increases accordingly [27]. The current intensity influences the generation rate of strong oxidizing radicals required for indirect oxidation reactions and the oxidation rate of organic matter on the electrode surface during direct reactions. When the current was too small, not enough highly oxidizing free radicals were produced to break the chemical bonds of oxytetracycline; thus, oxytetracycline had a low removal rate with little change in solution. According to the removal trend of oxytetracycline hydrochloride after electrolysis for 15 min in Figure 2, the removal effects of 0.400 and 0.550 A on oxytetracycline hydrochloride were not significantly different. Considering energy consumption, it was more reasonable to choose 0.4 A with a removal rate of 97.39%.

Plate spacing is one of the main factors affecting the removal of oxytetracycline. The plate spacing affects the field intensity of the solution between the plates, affecting the removal of pollutants. To explore the influence of the plate spacing on the removal of oxytetracycline, experiments were carried out with different plate spacings (1, 2, 3, and 4 cm), while other conditions (current: 0.4 A, Na_2_SO_4_ electrolyte concentration: 4 g/L, solution pH: 3) remained unchanged. According to the results presented in Figure 2, the removal at 5 min with a plate spacing of 1 cm was better than that using 2, 3, and 4 cm. At 10 min, the removal with an electrode spacing of 2 cm was better than that of 1, 3, and 4 cm. At 20 min, the maximum removal rate with a plate spacing of 2 cm was 97.39%. According to these results, a smaller plate spacing leads to greater electric field intensity, greater force and conductivity between electrodes, and thus better mass transfer effect in solution, and a higher removal rate of oxytetracycline at a given time. However, when the plate spacing is too small, a short circuit is more likely to occur, and the anode surface is also prone to passivation, increasing energy consumption. When the current is constant and the electrolyte concentration remains the same, a too large plate spacing increases the resistance between the plates. An increase in the plate spacing will decrease the field intensity experienced by the organic matter to be degraded in solution between the anode and cathode plates and will decrease the electrical conductivity, thus reducing the current efficiency and removal efficiency and increasing energy consumption.

The addition of electrolytes can increase the conductivity, and the concentration of electrolytes will affect the conductivity of the solution and the power consumption during pollutant removal. To study the effect of the electrolyte concentration on the removal of oxytetracycline, the concentration of the Na_2_SO_4_ electrolyte (2, 3, 4, 5 g/L) was changed in the experiment, while other conditions (current: 0.4 A, plate spacing: 2 cm, solution pH: 3) remained unchanged. As shown in Figure 2, the rate of oxytetracycline removal gradually increased as the dielectric concentration increased from 2 to 4 g/L. As the electrolyte concentration increased, the conductivity of the solution increased, and the electron transfer rate increased, not only decreasing the tank voltage but also improving the current effect. When the electrolyte concentration was 5 g/L, the oxytetracycline removal rate was higher than that with electrolyte concentrations of 2 and 3 g/L but lower than that with electrolyte concentration of 4 g/L. Thus, an increase in the electrolyte concentration within a certain range improves the conductivity of the solution and promotes the removal of pollutants. When the electrolyte concentration increases to a certain value, the ions in the electrolytic system reach saturation, and then the efficiency of the pollutant removal decreases.

In an electrolytic reaction system, the pH of the solution is also an important factor affecting the reaction. As a key factor, pH affects the degree of dissociation of the substances in solution and the electrical conductivity of the solution; furthermore, pH affects the formation rate, yield, and hydrogen and oxygen evolution of strong oxidizing intermediates, all of which affect the electrochemical reaction rate. To explore the influence of solution pH on the removal of oxytetracycline, experiments were carried out with different pH (3, 5, 7, 9), while other conditions (current: 0.4 A, Na_2_SO_4_ electrolyte concentration: 4 g/L, plate spacing: 2 cm) remained unchanged. As shown in Figure 2, the rate of oxytetracycline removal increased gradually with decreasing pH, and increased significantly when the pH of the solution gradually became acidic. The oxytetracycline removal rate after electrolysis for 20 min at pH 3 decreased to 98.35%.

### 3.2. Orthogonal Experiments for Determining Optimal Conditions

Orthogonal experiments are a method for the exploration of multiple factors and multiple levels of experiments [20]. According to the conclusion of the control variable method, reasonable experimental factors were selected to carry out the orthogonal experiments. The control variable method solves the problem of finding experimental conditions and factors that cannot be determined by orthogonal experiments and fully exploits the advantages of orthogonal experiments. The effects of current intensity, electrolyte concentration and solution pH on the degradation of oxytetracycline were investigated by orthogonal experiments to determine the best experimental conditions for oxytetracycline degradation. The factor level table is shown in Table 1.

The orthogonal experimental results and analysis designed by three factors and three levels are shown in Table 2.

As seen from Table 2, the optimal orthogonal experimental scheme was A3B2C2, in which the electrolysis current is 0.500 A, electrode spacing is 2 cm, concentration of Na_2_SO_4_ electrolyte is 4 g/L, and solution pH is 3. The rate of oxytetracycline degradation reached 99.02% after 20 min of electrolysis, which was higher than the 98.85% degradation rate of the A3B2C1 combination in the orthogonal experiment. Therefore, the optimal condition was A3B2C2. The range analysis showed that the order of the three factors affecting the degradation rate was A > B > C, and the current intensity had the greatest impact on the oxytetracycline degradation rate.

### 3.3. Characterization of the Ti/IrO_2_-Ta_2_O_5_ Anode

#### 3.3.1. SEM Characterization

**Figure 3:**
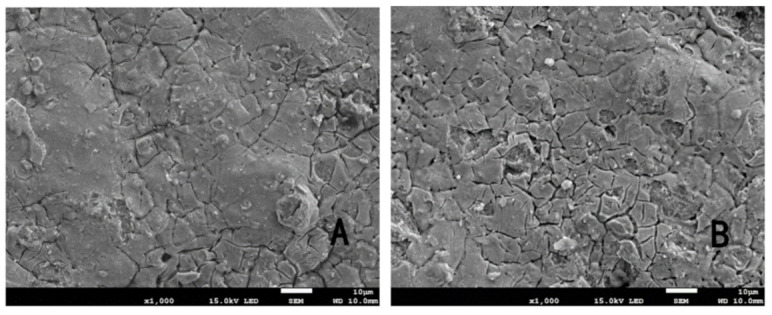
SEM of the Ti/IrO_2_-Ta_2_O_5_ electrode: (**A**) before use at 10 μm and (**B**) after use at 10 μm.

As shown in Figure 3, the electrode surface changed during the experiment. Prior to the experiment, the Ti/IrO_2_-Ta_2_O_5_ electrode had small cracks and raised particles on the surface. The cracks are due to the thermal stress between the interfaces of different substances during production [28]. This structure had the advantage that the oxide coating was tightly bound to the titanium substrate and provided a large number of reaction sites to facilitate the electrolytic reaction [29]. After 100 h of the experiment, the surface cracks of the electrode increased significantly. During the electrolysis, the electrolyte reached the surface of the titanium matrix through the cracks and gaps, giving rise to corrosion passivation and affecting the life of the electrode. Therefore, new methods should be considered to reduce the thermal stress between different substances so that the Ir-Ta oxide can form a better solid solution, the cracks between the substrate and coating can be avoided, and the binding force between the coating and substrate can be increased.

#### 3.3.2. EDS Characterization

**Figure 4:**
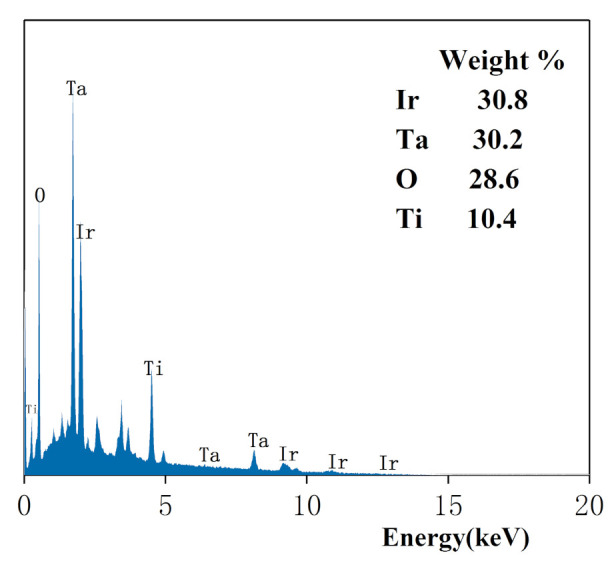
X-ray energy-dispersive spectroscopy (EDS) of the Ti/IrO_2_-Ta_2_O_5_ electrode.

As shown in Figure 4, the electrode exhibited multiple diffraction peaks under different voltage conditions. Based on these peaks, it was concluded that the rare earth Ir and Ta elements were present on the plate. The peak areas of Ir and Ta were large, and their weight percentages were 30.8 and 30.2%, respectively. In addition, the presence of O and Ti on the electrode surface was identified according to their peaks. The mass fractions of O and Ti were 28.6 and 10.4%, respectively. These results showed that Ir and Ta were well mixed on the plate surface. Thus, an Ir-Ta coating improves the catalytic performance and prolongs the service life of an electrode.

#### 3.3.3. XRD Characterization

**Figure 5:**
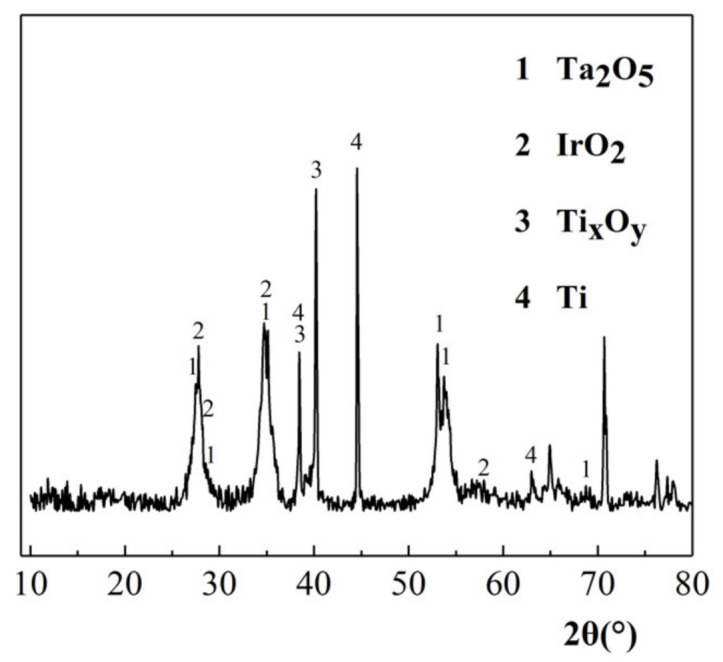
XRD of Ti/IrO_2_-Ta_2_O_5_ electrode.

As shown in Figure 5, the diffraction peaks of Ta_2_O_5_ (JCPDS 97-028-0397), IrO_2_ (JCPDS 97-008-4577), Ti (JCPDS 00-005-0682), and Ti oxide (JCPDS 97-000-0926 97-000-9646 97-000-0932) were observed in the XRD characterization results of the Ti/IrO_2_-Ta_2_O_5_ electrode. The diffraction peaks of Ta_2_O_5_ and IrO_2_ indicated that the coating material was fixed on the surface of the plate. The diffraction peaks of Ti and Ti oxides were due to the small cracks caused by the thermal stress between the different substances of the plate coating material. The prepared Ti/IrO_2_-Ta_2_O_5_ anode met the test requirements.

#### 3.3.4. Kinetics of the Electrochemical Degradation of Oxytetracycline

**Figure 6:**
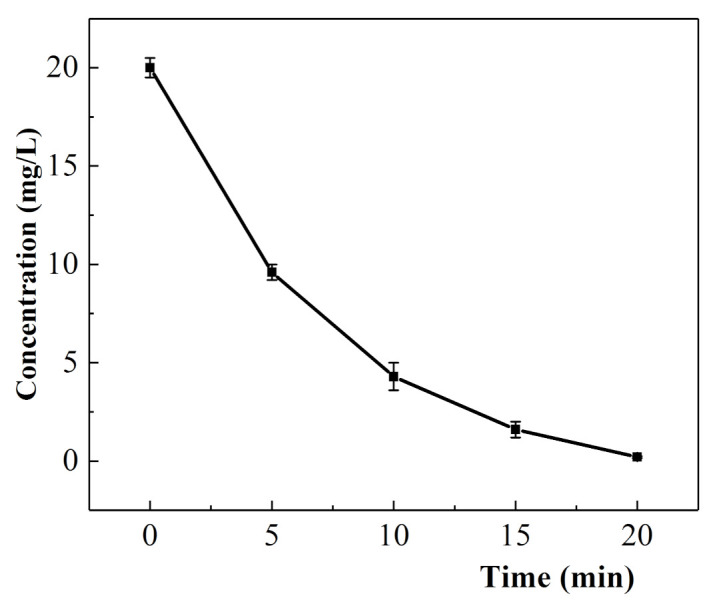
Concentration of oxytetracycline as a function of time.

Under the optimal reaction conditions (current: 0.5 A, plate spacing: 2 cm, Na_2_SO_4_ electrolyte concentration: 4 g/L, solution pH: 3), the oxytetracycline concentration changed over time, as shown in Figure 6. The concentrations of the oxytetracycline solutions at different times (*C_p_*), with time (*t*) as the abscissa and $c_{p}-c_{p0}$,$\;\ln\left(c_{p}/c_{p0}\right)$, $c_{p0}^{-1}-c_{p}^{-1}$ as the ordinate, were selected for dynamic calculation. In the three fitting curves, the first-order kinetic equation y = −0.22004x + 0.32002, $\ln\left(c_{p}/c_{p0}\right)$ showed a good linear relationship with t, and the R^2^ value of 0.92669 for this equation was the largest. The parameters of the fitting curve are shown in Table 3. These results indicated that the reaction rate of the electrochemical degradation of oxytetracycline was related to the concentration of the substance involved in the reaction under the optimal conditions, and the reaction was in accordance with first-order kinetics. The reaction rate between the 20 mg/L oxytetracycline hydrochloride solution and active particles was 0.22004 min^−1^.

### 3.4. Degradation Pathway and Product Analysis of Oxytetracycline

**Figure 7:**
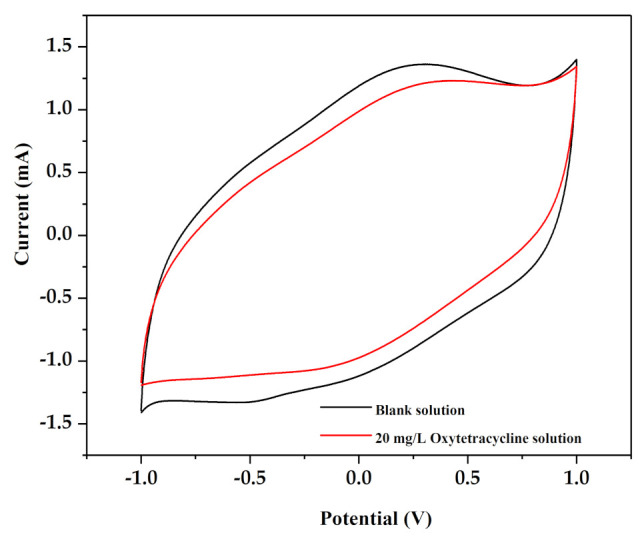
Cyclic voltammetry (CV)curve of Ti/Ta2O5-IrO2 electrode in blank solution and 20 mg/L oxytetracycline solution.

Figure 7 shows the cyclic voltammetry curves of the Ti/IrO_2_-Ta_2_O_5_ electrode in a blank solution (Na_2_SO_4_ solution) and a 20 mg/L oxytetracycline solution. The cyclic voltammetry (CV) curve of the 20 mg/L oxytetracycline solution had the same shape as the CV curve of the blank solution, and no new peaks were found, indicating that there was no direct electron transfer on the Ti/IrO_2_-Ta_2_O_5_ electrode. The electrochemical oxidation of organic matter on the anode surface was divided into direct oxidation and indirect oxidation. The absence of a new peak of the anode current during the forward scan implied that the oxidation reaction did not directly occur on the anode surface, and the degradation of organic matter was mainly carried out through indirect oxidation [30]. Therefore, in this experiment, oxytetracycline was not oxidized directly on the anode surface but rather was decomposed by the active substances (such as •OH) in the solution.

The cyclic voltammetry test results showed that oxytetracycline was not directly oxidized on the anode surface but may be decomposed by oxidants, such as hydroxyl radicals (•OH) in the solution. To verify this hypothesis, hydroxyl radicals produced during the reaction were captured by a free radical scavenger, and the generation mechanism of free radicals was investigated.

Common free radical-scavenging agents include tert-butanol, CO_3_^2−^, HCO_3_^−^, etc. In this experiment, tert-butanol was selected and added as the free radical-scavenging agent to conduct a control experiment to verify the above conjecture. The reaction of tert-butanol with hydroxyl radicals generates an inert radical, namely, $CH_{2}C{\left(CH_{3}\right)}_{2}OH$. The reaction rate constant K = 6.0 × 10^8^ mol•L^−1^•S^−1^. After the reaction of tert-butanol with hydroxyl radicals, the activity and oxidizing ability of hydroxyl radicals decreased.

**Figure 8:**
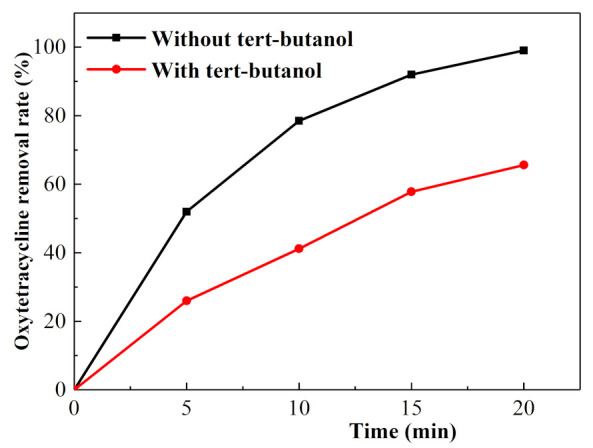
Degradation curve of oxytetracycline.

Under the optimal experimental conditions, the removal comparison curve of the oxytetracycline solution without tert-butanol and the oxytetracycline solution with a tert-butanol concentration of 30 mg/L is shown in Figure 8. The removal rate of oxytetracycline solution with tert-butanol was lower than that of the oxytetracycline solution without tert-butanol, and the removal rates of the oxytetracycline solution without tert-butanol and the oxytetracycline solution with tert-butanol were 93.74 and 59.30%, respectively, after 20 min of electrochemical treatment with a 20 mg/L oxytetracycline solution. The addition of tert-butanol had a clear inhibitory effect on the removal of oxytetracycline. Therefore, the oxytetracycline removal process was related to •OH. Thus, a large amount of •OH was produced in the electrolysis process that could effectively destroy the oxytetracycline structure.

Therefore, it can be concluded that the indirect oxidation reaction represented by Equations (3) and (4) occurred on the surface of the dimension stable anode electrode during electrolysis. MOx represents the metal oxide coating of the Ti/IrO_2_-Ta_2_O_5_ electrode.

(3) $$MO_{x}+H_{2}O\to MO_{x}\left(\cdot OH\right)+H^{+}+e^{-}$$

(4) $$MO_{x}+MO_{x}\left(\cdot OH\right)\to MO_{x}+O_{2}+2H^{+}+2e^{-}$$

### 3.5. Degradation Product Analysis of Oxytetracycline

Since tetracycline organics are conjugated compounds with two chromophiles, they have strong ultraviolet absorption in the visible range [31]. The oxytetracycline solution had two strong ultraviolet absorption peaks near 275 and 355 nm.

**Figure 9:**
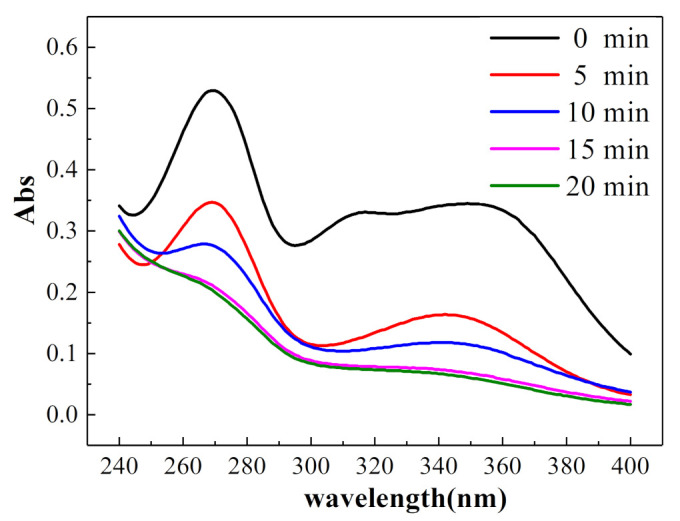
UV–Vis absorption spectra of 20 mg/L oxytetracycline solution.

In Figure 9, the UV–Vis absorption spectra of 20 mg/L oxytetracycline solution after electrolysis for 0, 5, 10, 15, and 20 min under the optimal experimental conditions are shown with the progress of the electrolysis reaction, the absorption intensities of the peaks near 275 and 355 nm gradually weakened, and the intensity of the peak at 275 nm decreased strongly. It is speculated that some functional groups were destroyed due to the action of hydroxyl radicals. Furthermore, it is speculated that the hydroxyl group on carbon No. 10 of oxytetracycline HCl was easily oxidized to form C=O bonds in a strong oxidizing system; thus, the acidity decreased. However, after 20 min of electrolysis, the UV absorption peaks of the two functional groups were still present, indicating that only a part of the structure of the two functional groups was damaged.

Some functional groups and structures of oxytetracycline could be destroyed through the production of a large amount of •OH during the electrochemical degradation of oxytetracycline. According to the above hypothesis of the production of some small organic molecules or difficulty to degrade organic matter, liquid chromatography–mass spectrometry (LC-MS) was used to analyze the oxytetracycline solution after electrolytic treatment for 20 min under optimal conditions. The degradation products were analyzed according to the mass–charge ratio, molecular weight, and oxytetracycline structure.

**Figure 10:**
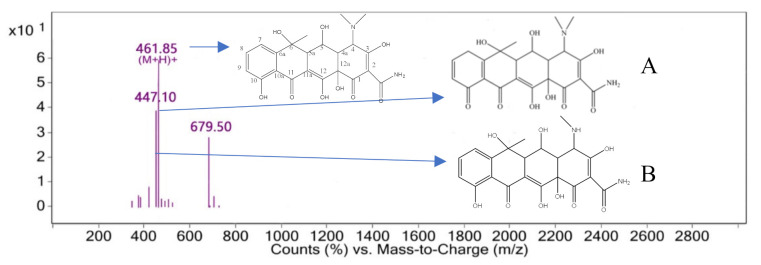
Mass spectrum at 3.892 min.

The characteristic peak retention time of 3.892 min was obtained by mass spectrometry in the mass–charge ratio range of 0–2800, as shown in Figure 10. The mass–charge ratio M/Z of 461.85[M+H]+ was assigned to oxytetracycline (the remaining oxytetracycline molecules present in the solution), as shown in Figure 10. Since the concentration of oxytetracycline was very low after 20 min of electrolysis, the ultraviolet absorption peak at 275 nm was significantly reduced, and the pH of the solution decreased, it is speculated that this characteristic peak was more likely to be compound 1 generated by the oxidation of the hydroxyl group on carbon No. 10 to the C=O bond, as shown in Figure 10A. The substance with an M/Z of 447.10 was 14 atomic masses less than M/Z 461.85[M+H]+, and it is hypothesized that this result was due to the replacement of the methyl group of the dimethylamino on carbon No. 4 by hydrogen to obtain compound 2, as shown in Figure 10B.

**Figure 11:**
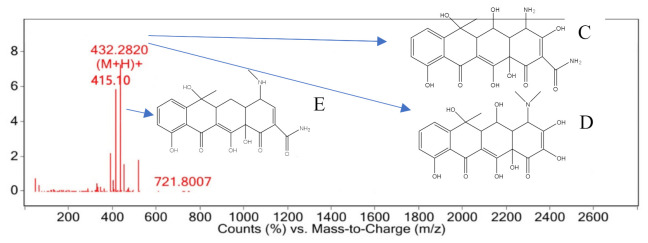
Mass spectrum at 6.021 min.

The characteristic peak retention time of 6.021 min was determined by mass spectrometry in the mass–charge ratio range of 0–2600, as shown in Figure 11. The M/Z of 432.28[M+H]+ that may not be well matched to M/Z 433 or M/Z 434 is hypothesized based on the formation process of compound 2 and the structural formula of oxytetracycline. The methyl group on dimethylamine at carbon No. 4 was easily replaced by hydrogen to form compound 2. If both methyl groups were replaced, a substance with an M/Z of 433 may be obtained, namely, compound 3, as shown in Figure 11C. The substance with an M/Z of 434 may be compound 4 with a molecular weight of 434 that is speculated to be formed by amide bond fracture on carbon No. 2 and hydroxyl radical combination; its structure is shown in Figure 11D. The substance with an M/Z of 415.10 may be based on compound 2, with one hydroxyl group removed from carbon No. 3 and carbon No. 5 to obtain compound 5, as shown in Figure 11E.

**Figure 12:**
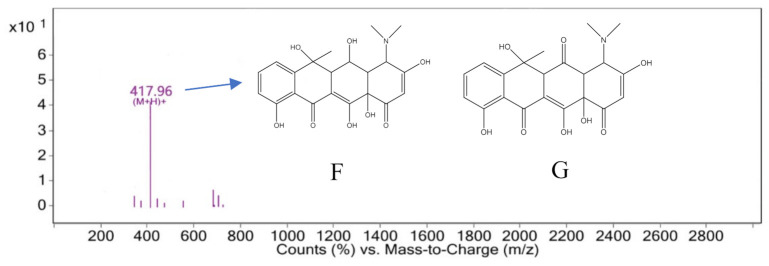
Mass spectrum at 3.162 min.

The characteristic peak retention time of 3.162 min was analyzed by mass spectrometry in the mass–charge ratio range of 0–2800, as shown in Figure 12. The M/Z of 417.96[M+H]+ that may correspond to compound 6 with a molecular weight of 417 and its molecular weight differed by 16 from that of compound 3 with a molecular weight of 433. It is speculated that the carbon No. 2 of oxytetracycline did not bind hydroxyl radicals but was reduced to compound 6, as shown in Figure 12F. Compound 6 was easily oxidized as the hydroxyl group on carbon No. 5 may be oxidized to a carbonyl, resulting in compound 7 with a molecular weight of 415. Furthermore, it may be another substance from Figure 11 with an M/Z of 415.10, and its structure is shown in Figure 12G.

**Figure 13:**
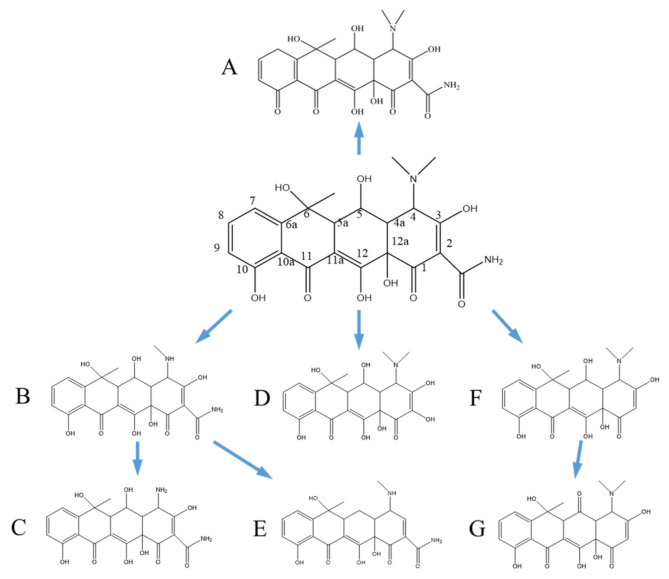
Diagram of degradation process of oxytetracycline. (**A**–**G**) were the compounds produced by the degradation process, respectively.

Based on the conjecture analysis of the degradation products of oxytetracycline, a possible degradation pathway was speculated, as shown in Figure 13.

The hydroxyl group on carbon No. 10 of oxytetracycline was oxidized into a C=O bond to form compound A. The methyl group on dimethylamine at carbon No. 4 was replaced by hydrogen to produce compound B, and if another methyl group was also substituted, compound C was obtained. On the basis of compound B, one hydroxyl group was removed from carbon No. 3 and carbon No. 5 to obtain compound E. The amide bond on oxytetracycline carbon No. 2 was broken and combined with hydroxyl radicals to form compound D. However, if the oxytetracycline part of carbon No. 2 did not bind hydroxyl radicals, it was reduced to compound F. The hydroxyl group on carbon No. 5 of compound F was more easily oxidized to a carbonyl to form compound G.

Substituents determine a series of chemical properties such as drug resistance and biotoxicity. Therefore, the substitution reaction of oxytetracycline in the degradation process changed its chemical properties; this may improve the biodegradability of the compound itself. Compared with previous studies on the electrochemical degradation of tetracycline hydrochloride, the Ti/Ta_2_O_5_-IrO_2_ anode has the potential for further research in the degradation of antibiotic pollutants.

## 4. Conclusions

The electrochemical oxidation of oxytetracycline using a Ti/IrO_2_-Ta_2_O_5_ anode is feasible. Orthogonal experiments were carried out after a single-factor experiment on the factors affecting the degradation of oxytetracycline; additionally, kinetic calculations were carried out. The optimal conditions were determined to be as follows: the current was 0.500 A, the plate spacing was 2 cm, the concentration of Na_2_SO_4_ electrolyte was 4 g/L, and the solution pH was 3. After the test, a few cracks were generated on the surface of the plate. The change in the rate of oxytetracycline removal after three repeated experiments was within the error tolerance range. Since no destructive tests have been performed on the plates, the lifetime of the plates should be studied further. The three factors with the greatest influence on the degradation rate of oxytetracycline were in the order of: current intensity > electrolyte concentration > solution pH. The process of oxytetracycline by the electrochemical method agreed with the first-order kinetic reaction. The electrochemical tests of the degradation process of oxytetracycline showed that there was no direct electron transfer on the Ti/Ta_2_O_5_-IrO_2_ electrode; rather, hydroxyl radicals were generated, thereby promoting an indirect electrochemical reaction. The oxytetracycline degradation products were analyzed by full-band UV scanning and LC-MS detection, and seven possible compounds and five possible degradation pathways were proposed.

## Figures and Tables

**Table 1:** Factor level table of the orthogonal experiments.

Factors	Level		
	Level 1	Level 2	Level 3
A: Current intensity (A)	0.300	0.400	0.500
B: Electrolyte concentration (mg/L)	3	4	5
C: Solution pH	2	3	4

**Table 2:** Orthogonal experimental results and range analysis table.

Serial Number	A	B	C	Degradation Rate (%)
1	1	1	1	87.38
2	1	2	2	97.03
3	1	3	3	83.57
4	2	1	2	93.85
5	2	2	3	97.51
6	2	3	1	98.80
7	3	1	3	98.04
8	3	2	1	98.85
9	3	3	2	98.23
K1	89.327	93.090	95.010	/
K2	96.720	97.797	96.370	/
K3	98.373	93.533	93.040	/
R	9.046	4.707	3.330	/

**Table 3:** Dynamic fitting curve parameters at various plate spacings.

Reaction Order	Kinetic Equation	Fitting Curve	R^2^
First-order reaction	$\ln\left(c_{p}/c_{p0}\right)=-k_{t}+b$	y=−0.22004x+0.32002	0.92669
